# Effectiveness of Acupressure on the Taichong Acupoint in Lowering Blood Pressure in Patients with Hypertension: A Randomized Clinical Trial

**DOI:** 10.1155/2016/1549658

**Published:** 2016-10-10

**Authors:** Gan-Hon Lin, Wei-Chun Chang, Kuan-Ju Chen, Chen-Chen Tsai, Sung-Yuan Hu, Li-Li Chen

**Affiliations:** ^1^Department of Nursing, Taichung Veterans General Hospital, 1650 Taiwan Boulevard Sect. 4, Taichung 40705, Taiwan; ^2^Cardiovascular Center, Taichung Veterans General Hospital, 1650 Taiwan Boulevard Sect. 4, Taichung 40705, Taiwan; ^3^Department of Traditional Chinese Medicine, Taichung Veterans General Hospital, 1650 Taiwan Boulevard Sect. 4, Taichung 40705, Taiwan; ^4^Chander Clinic, No. 128, Sec. 3, Dongxing Rd., Taichung 40356, Taiwan; ^5^Department of Emergency Medicine, Taichung Veterans General Hospital, 1650 Taiwan Boulevard Sect. 4, Taichung 40705, Taiwan; ^6^School of Medicine and Institute of Medicine, Chung Shan Medical University, No. 110, Sec. 1, Jianguo N. Rd., Taichung 40201, Taiwan; ^7^School of Nursing, College of Health Care, China Medical University, 91 Hsueh-Shih Road, Taichung 40402, Taiwan; ^8^Department of Nursing, China Medical University Hospital, 2 Xue Shi Road, Taichung 40402, Taiwan

## Abstract

*Objectives*. To evaluate the effectiveness of acupressure on the Taichong acupoint in lowering systolic and diastolic blood pressure (BP) in hypertensive patients.* Methods*. Eighty patients with hypertension attending a cardiology outpatient department in central Taiwan were included in this randomized clinical trial. Acupressure was applied to the Taichong acupoint in the experimental group (*n* = 40) and to the first metatarsal (sham acupoint) in the control group (*n* = 40). Blood pressure was measured by electronic monitoring before and immediately 15 min and 30 min after acupressure.* Results*. The average age of the experimental and control participants was 59.3 ± 9.2 years and 62.7 ± 8.4 years, respectively. The two groups were similar for demographics and antihypertensive drug use. Mean systolic and diastolic BP in the experimental group decreased at 0, 15, and 30 min after acupressure (165.0/96.3, 150.4/92.7, 145.7/90.8, and 142.9/88.6 mmHg); no significant changes occurred in the control group. There was a significant difference in systolic and diastolic BP between the experimental and control groups immediately and 15 and 30 min after acupressure (*p* < 0.05).* Conclusion*. Acupressure on the Taichong acupoint can lower BP in hypertensive patients and may be included in the nursing care plan for hypertension. However, additional studies are needed to determine the optimal dosage, frequency, and long-term effects of this therapy.

## 1. Introduction

Hypertension is a common disease and a major risk factor for coronary artery ischemia and stroke. The incidence of hypertension is projected to reach 29.2% (1.56 billion) by 2025 [1]. In the meantime, approximately 8 million people die from complications of hypertension every year. The prevalence of hypertension is 29.6% in the US population aged older than 18 years [2] and 22.6%–24% and those aged older than 15 years in Taiwan [3]. It is known that blood pressure (BP) is positively correlated with the risk of heart attack, heart failure, stroke, and renal disease [4]. Hypertension also ranked eighth among the leading causes of death in Taiwan in 2014. Thus, prevention and treatment of hypertension are an issue of international medical concern and a challenge faced by all medical professionals.

Effective control of hypertension can decrease the incidence of heart attack and stroke. The World Health Organization (2013) recommends combining nonpharmacological treatment with antihypertensive drugs to control BP in patients with hypertension [5]. Despite combined treatment of hypertension, including traditional Chinese and conventional medicine, surveys have found that a large proportion of patients with hypertension do not achieve adequate BP control [2, 6, 7]. Thus, effective BP control should be the main objective in the ongoing effort to prevent and treat hypertension.

Taiwan has now integrated traditional Chinese medicine into the national healthcare system [8]. In addition to Chinese herbs and conventional medicines, traditional Chinese medical acupuncture is also used in the treatment of hypertension [9]. A survey in Taiwan found that the population often used a combination of conventional and traditional Chinese medicine [10]; 62.5% of those surveyed used traditional Chinese medicine at least once [11] and 81% had used complementary alternative medicine (CAM) in the previous year. The most commonly used forms of CAM include Gua Sha, cupping, tuina, and massage (45%) [8]. Thus, it is clear that there is a high degree of acceptance of acupoint methods and meridian treatment among the Taiwanese.

Although Kaneko et al. reported that Taichong acupuncture (LR3) did not lower BP in their study patients [12], other studies in both animals and humans have suggested that acupuncture on the Taichong acupoint is effective in the treatment of hypertension [13–21]. The effect of acupuncture on the Taichong acupoint in the treatment of hypertension is rapid in onset, lowering systolic and diastolic BP within 20–30 min [13, 14]. Acupuncture and acupressure are based on the meridian system of traditional Chinese medicine and use fingers or needles to stimulate acupoints by various techniques to achieve the same therapeutic effect [22].

Previous research has examined the combined effect of tuina and acupressure on BP control [23]. However, no experimental studies have been reported on the BP-lowering effects of Taichong acupressure. Thus, the purpose of this study was to explore the ability of acupressure on the Taichong acupoint to lower BP in outpatients with hypertension. The results of this study could provide nursing staff with a further therapeutic option when managing patients with hypertension. Further, nurses are well placed to teach patients and/or their family members to perform this massage technique at home to enhance the quality of care available for patients with hypertension.

## 2. Materials and Methods

### 2.1. Design

We used a randomized controlled trial using repeated measures.

### 2.2. Participants

The study population was drawn from patients attending the cardiology outpatient department at a medical center in central Taiwan between August 2012 and January 2013. The study inclusion criteria were as follows: a diagnosis of primary hypertension by a physician; a baseline systolic BP of 150–180 mmHg; age 40–75 years; willingness to participate in the study and sign the consent form. The exclusion criteria were as follows: suspected acute stroke, chest tightness, or pain of cardiac origin; ingestion of short-acting antihypertensive medication within 2 h prior to the beginning of the study; skin damage at the Taichong acupoint or injury of the lower extremities; body temperature > 37.5°C (99.5°F); dyspnea; or pregnancy.

Patients were screened by physicians in the outpatient clinic to determine if all eligibility criteria were met. The participants were moved to a quiet discussion room in order to avoid the noisy atmosphere in the clinic and to decrease the risk of white-coat hypertension, which could affect the study results [24]. Study participants were asked to sit on a sofa chair with back support in the discussion room for the duration of the study. The researcher selected a sealed envelope depending on the patient's sex to determine group allocation. It took approximately 15–20 min to explain the consent form and secure written informed consent from each study participant. This time interval had the advantage of buffering any transient changes in BP that may have occurred as a result of movement between the clinic and the discussion room.

### 2.3. Intervention

**Figure 1:**
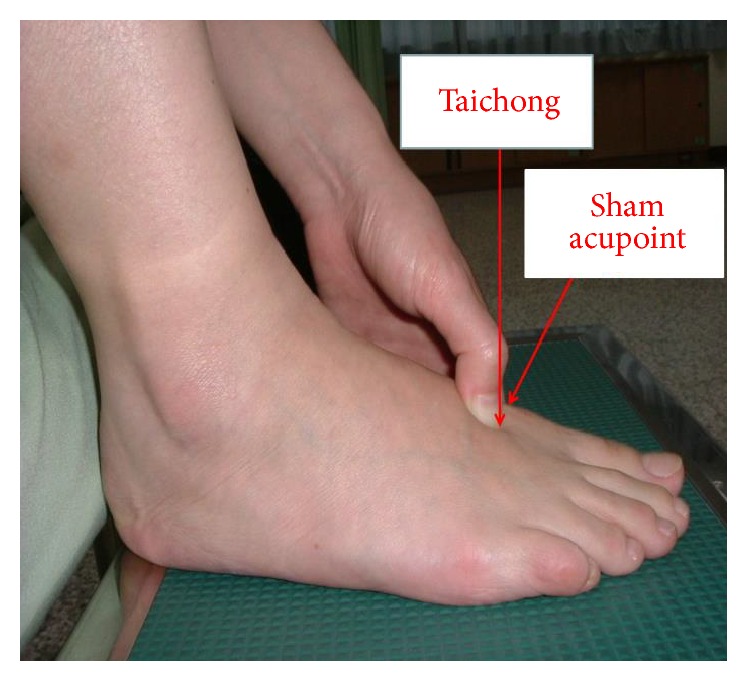
Locations of Taichong acupoint and sham acupoint.

The intervention was performed in all study subjects by the same researcher. The acupressure technique and procedures used were identical in the two groups; the only difference was the location of the acupressure points. In the experimental group, acupressure was applied to the Taichong acupoint of the right foot (located on the dorsum of the foot in the distal hollow at the junction of the first and second metatarsal bone, Figure 1). In the control group, acupressure was applied to a sham acupoint approximately 1 inch from the medial side of the Taichong acupoint on the first metatarsal. In both groups, the thumb was used to apply pressure to the acupoint in a perpendicular manner. Pressure was applied and held for 5 seconds (sec) and then released for 1 sec. This hold-release pattern was repeated 30 times over the course of 3 min. The pressure applied was approximately equivalent to 3 kg [25].

### 2.4. Sample Size

The sample size was calculated from the findings of a pilot study that included 8 participants (4 men, 4 women). The mean and standard deviation for the difference in systolic BP before and 15 min after acupressure between the two groups (effect size) were entered into G Power v.3.1.2 software [26] and set to* t*-test. A priori analysis was performed to calculate the sample size with the following input parameters: two tails, *α* error probability = 0.05, and power (1 − *β* error probability) = 0.8. The required sample size was calculated to be 60. Using an estimated 20% drop-out rate, we would need 80 subjects. Forty subjects were recruited for the experimental group and 40 for the control group. All participants enrolled completed the study.

### 2.5. Randomization

In this study, a gender-stratified random assignment, conducted by nonteam members prior to the beginning of the study, was used to exclude the potential effect of sex on BP and acupressure [3, 27–30]. The randomization process included the following: (1) 20 strips of paper labeled “control group” and 20 labeled “experimental group” being placed individually in opaque envelopes; (2) the envelopes being placed in a box labeled “male group” and each qualifying male participant receiving an envelope randomly picked from the box; (3) each participant being assigned to the experimental group or control group depending on the labeled strip in the envelope. For female participants, the random assignment steps were identical to those used in the male participants.

### 2.6. Measures

#### 2.6.1. Validity and Reliability of Participant Data Record Sheet

The following data were recorded: basic information for each participant, BP prior to and following acupressure, medical conditions, current medications, and any other factors that may influence BP. Next, a 6-person panel comprising Western physicians, Chinese medicine physicians, and nurse specialists performed content validity tests. The content validity index for all items ranged from 0.83 to 1.00.

#### 2.6.2. Validity and Reliability of Electronic BP Monitor

An oscillometric electronic BP monitoring device (CAS740, CAS Medical Systems, Inc., Branford, CT, USA) was used to measure BP prior to and following acupressure. According to the manufacturer, the device has a precision of ±5 mmHg and a standard deviation not greater than 8 mmHg. The accuracy of the electronic BP monitor was calibrated and tested regularly by the hospital medical engineering personnel. The reliability of the monitor was tested using the same monitor and measurement method in the study site. The monitor was set to automatically measure BP four times at 1-minute intervals and the test was conducted on 5 subjects. Thereafter, the coefficient of variation was 1.1%–2.1% and 1.3%–4.1% for systolic and diastolic BP, respectively.

#### 2.6.3. Validity and Reliability of Digital Baby Scale

The Digital Baby Scale (Model 727, Seca GmbH & Co. KG, Hamburg, Germany) used in this study had a precision of ±2 g and was used to train researchers to apply acupressure steadily and effectively. The training regime was performed over 2 consecutive weeks. The researcher was trained to apply pressure in a perpendicular manner with the right thumb to the middle of the surface of the Digital Baby Scale 30 times consecutively until 3 kg of pressure could be consistently applied and the error kept within 0.5 kg.

#### 2.6.4. Validity and Reliability of Acupressure Staff

Acupressure staff received 180 h of training in traditional Chinese medicine nursing courses. The training included meridian and acupoint theory as well as practical sessions. Prior to starting the study, the researcher was instructed on correct acupressure skills and the location of the Taichong acupoint by two Chinese physicians.

### 2.7. Data Collection

Personal information and BP were recorded for each participant by the same researcher. The effectiveness of acupressure was evaluated by measuring BP in the right upper arm [31] before and immediately after acupressure and 15 and 30 min later. Participant information was collected by verbal inquiry and a search of the medical records while waiting for BP to be measured.

### 2.8. Data Analysis

The statistical analysis was performed using SPSS version 22.0 for Windows (IBM Corp., Armonk, NY, USA). The frequency, percentage, mean, and standard deviation were used as descriptive statistics. The chi-square test and independent* t*-test were used to evaluate the homogeneity of the baseline demographics of participants in both groups and to evaluate the difference in BP in both groups before and after the acupressure intervention. The paired* t*-test was used to evaluate the difference in BP at baseline and after intervention. The linear mixed-effect model for repeated measures was used to analyze the effect of the intervention on BP under conditions where confounding factors such as gender, age, and medications were controlled.

### 2.9. Ethical Considerations

The research protocol was reviewed and approved by the institutional review board of Taichung Veterans Hospital in central Taiwan (number CF12105). Before recruitment, the study protocol was described to the subjects in detail, after which they signed consent forms. Participants were informed that they could withdraw from the study at any time and that withdrawal would not affect their treatment. After completion of the study, the researcher taught the controls how to locate the Taichong acupoint and apply self-acupressure.

## 3. Results

### 3.1. Participant Information

**Figure 2:**
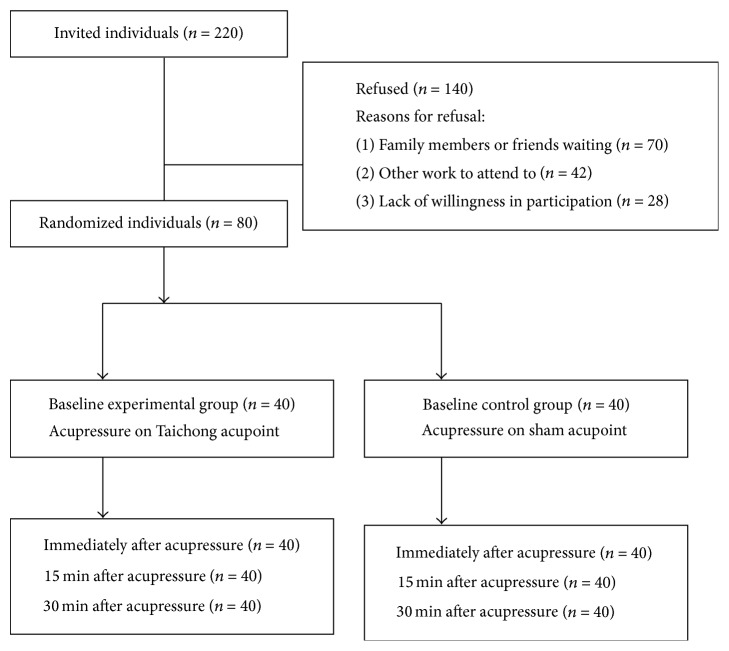
Flow of the study.

This study was conducted between August 2012 and January 2013. Of the 220 patients who met the inclusion and exclusion criteria, 80 agreed to participate and completed the study (Figure 2). The reasons for refusal to participate included the following: family members or friends were waiting to take the patient home (*n* = 70, 50%); other work to attend to (*n* = 42, 30%); and lack of interest or unwillingness in participation (*n* = 28, 20%). Most patients who agreed to participate were very interested in the aim of the study and asked for more information about acupressure, which may explain why there were no withdrawals from the study.

### 3.2. Distribution and Analysis of Demographics and Factors Affecting BP in Both Groups

Table 1 shows the distribution and statistical analysis of demographics and factors affecting BP in the experimental group (*n* = 40, mean age 59.25 ± 9.19 years) and control group (*n* = 40, mean age 62.70 ± 8.41 years). The majority of participants in both groups were on regular medication, and 45% of participants in each group had taken medication within the 8 h prior to participating in the study. The baseline systolic BP in the experimental group (164.98 ± 16.72 mmHg) was significantly (*p* < 0.05) higher than in the control group (154.80 ± 14.99 mmHg). There was no significant difference (*p* > 0.05) between these two groups with regard to diastolic BP or demographic parameters affecting BP at baseline.

### 3.3. Comparison of Effectiveness Characteristics of Acupressure in the Experimental and Control Groups

**Figure 3:**
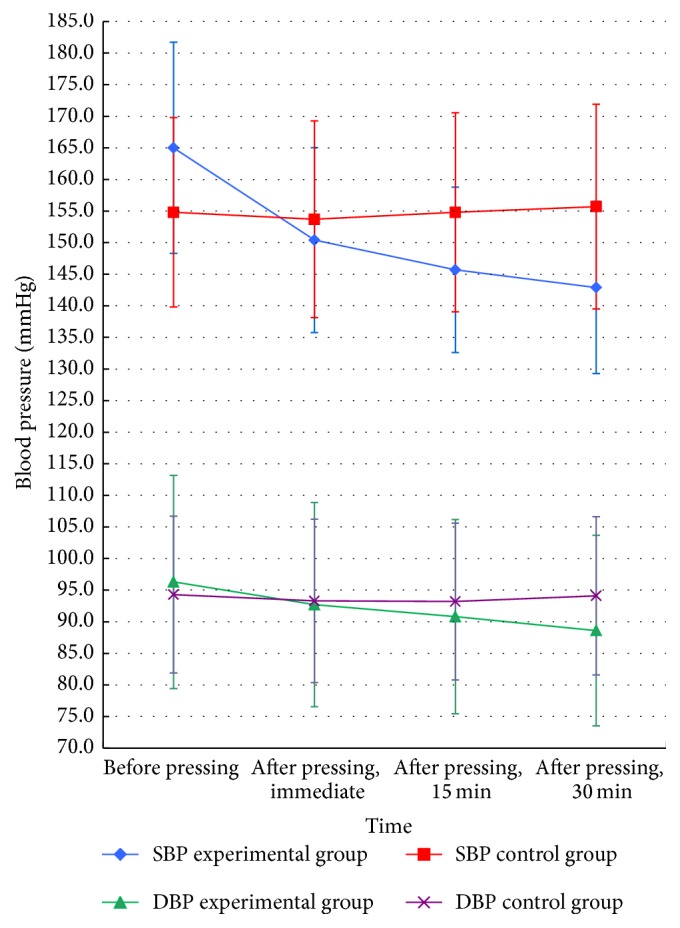
Trends of change in mean (± standard deviation) SBP and DBP before and after acupressure. DBP, diastolic blood pressure; SBP, systolic blood pressure.

The mean systolic and diastolic BP in the experimental group tended to decrease after acupressure, but there were no significant BP changes in the control group (Figure 3).  Table 2 shows the changes in BP prior to and following acupressure as well as a comparison of the effectiveness characteristics of acupressure between the two groups. In the experimental group, systolic BP decreased by a mean of 14.6, 19.3, and 22.1 mmHg and diastolic BP decreased by a mean of 3.6, 5.5, and 7.4 mmHg when compared with the control group at 0, 15, and 30 min after application of acupressure. A paired* t*-test demonstrated that the difference in BP before and after intervention was significantly different in the experimental group (*p* < 0.05), but not so in the control group (*p* > 0.05). An independent* t*-test comparing changes in BP in both groups at baseline and at different time points following acupressure showed that the decrease in systolic and diastolic BP in the experimental group was significantly greater than in the control group (*p* < 0.05).

A linear mixed-effect model for repeated measures was used to analyze the influence of group (experimental or control), time between measures (time 1 [interval between preacupressure and immediately after acupressure]; time 2 [interval between preacupressure and 15 min after acupressure]; time 3 [interval between preacupressure and 30 min after acupressure]), and their interaction, and gender, age, and medication use on the changes in systolic and diastolic BP. Among the factors affecting the change in systolic BP, the groups as well as interactions between groups and time interval between measures were significantly different (*p* < 0.05). In contrast, factors such as time between measures, gender, age, and medication use were not significantly different (*p* > 0.05; Table 3). Among the factors affecting change in diastolic BP, gender, age, interactions between groups, and time between measures were significantly different (*p* < 0.05). Group, time between measures, and medication use were not significantly different (*p* > 0.05, Table 4). The effectiveness of acupressure in lowering systolic and diastolic BP in the experimental group was significantly different from that in the control group after adjusting for confounding factors, including gender, age, and medication use. Specifically, the decrease in systolic and diastolic BP immediately and 15 min and 30 min after acupressure in the experimental group was greater than in the control group.

## 4. Discussion

The results of this study demonstrated that acupressure on the Taichong acupoint was much more effective than acupressure on a sham acupoint in lowering systolic and diastolic BP in patients with hypertension. Further, acupressure on the Taichong acupoint immediately lowered BP, and the lowering effect lasted for at least 30 minutes. Therefore, nurses could teach patients with hypertension and their family members how to use acupressure on the Taichong acupoint as a self-care strategy. Further studies are needed to establish the effectiveness of this therapy in the real-world setting, including dosage, compliance, and long-term results.

In this randomized study, the distribution of gender, age, smoking status, history of hypertension, medication use, and preacupressure diastolic BP of participants in both groups were similar. The baseline systolic BP in the experimental group was significantly higher than in the control group. However, the influence of baseline BP on changes in postacupressure BP could be adjusted using the linear mixed-effect model for repeated measures and by setting the preacupressure BP value as the baseline [32, 33].

Previous studies have shown that acupuncture at the Taichong acupoint can lower BP [13, 14, 17, 34]. The average decrease in systolic and diastolic BP in the present study was similar to that obtained after acupuncture [14, 35]. In traditional Chinese medicine, hypertension belongs to the categories of “dizziness” and “headache” [9, 36]. The Huangdi Neijing states that “all wind and dizziness disorders belong to the liver.” The Taichong acupoint is the yuan (source) acupoint of the liver meridian, and it has been documented in the literature that stimulation of the Taichong acupoint could spread liver Qi [9, 37]. Yuan (source) points are meridian points where the vital energy (yuan Qi) of the zang-fu organs passes and stays. These points are implicated in disease related to the five Yin organs and are responsible for the regulation of yuan Qi. Puncturing the yuan (source) points can stimulate the vital energy of the regular meridians, regulating the functional activity of the internal organs [38, 39]. Therefore, acupressure on the Taichong acupoint may stimulate liver function to facilitate the smooth flow of (liver) qi throughout the body, thereby lowering systolic and diastolic BP. However, further studies are needed to explore the mechanism of acupressure.

Previous studies have shown that the ability of acupuncture to lower BP can last for up to 150 minutes [35]. In this study, although BP was monitored for only 30 min after acupressure, the changes in BP before and after acupressure in the experimental group indicated a continuing downward trend of BP at 30 min after acupressure. Further studies are needed to identify the duration of effect of acupressure with regard to lowering BP to establish an appropriate time interval between each application of acupressure on the Taichong acupoint.

In this single-blind study, all intervention steps were performed by one researcher to ensure consistency. However, certain researcher-related confounders could not be entirely avoided. To minimize confounding and increase the operational precision of the researcher, the following measures were adopted: (1) development of a unified experimental protocol and explanatory information before the study; (2) minimization of measurement errors by using the same electronic BP monitor and avoiding operator error; and (3) participation in a training program to ensure the researcher could apply acupressure steadily and effectively. Blood pressure was measured only once at each time point in this study, which will inevitably result in some errors and variability, but these errors occurred randomly at the preacupressure and postacupressure time points. It should be pointed out that the generalizability of the results of this study to hypertensive patients younger than 40 years or older than 75 years is limited. In this study, because BP was monitored for only up to 30 min after application of acupressure, the effectiveness of acupressure in lowering BP beyond 30 min cannot be determined. Therefore, the effective duration of acupressure has yet to be determined.

## 5. Conclusion

Acupressure is one of the methods used in traditional Chinese medicine for the prevention and treatment of disease. It is a simple, noninvasive technique that nurses can perform independently. These findings suggest that acupressure on the Taichong acupoint could lower systolic and diastolic BP in patients with hypertension for at least 30 min. Further studies are needed to establish the effectiveness of this therapy in the real-world setting, including dosage, compliance, and long-term results.

## Figures and Tables

**Table 1:** Distribution and analysis of demographic characteristics and factors affecting blood pressure in both groups.

Variables	Experimental group	Control group	*χ* ^2^/*t*	*p* value
	(*n* = 40)	(*n* = 40)		
	*n* (%)/mean ± SD	*n* (%)/mean ± SD		
Gender			<0.001	1
Male	20 (50.0)	20 (50.0)		
Female	20 (50.0)	20 (50.0)		
Smoking status			0.392	0.531
Yes	7 (17.5)	5 (12.5)		
No	33 (82.5)	35 (87.5)		
Regular medication use			0.549	0.459
Yes	27 (67.5)	30 (75.0)		
No	13 (23.5)	10 (25.0)		
Medication taken within 8 h			21.2	1
Yes	18 (45.0)	18 (45.0)		
No	22 (55.0)	22 (55.0)		
Age (years)	59.25 ± 9.19	62.70 ± 8.40	−1.752	0.084
Years on medication	4.30 ± 5.17	6.28 ± 7.36	−1.389	0.169
History of hypertension (years)	7.08 ± 5.69	7.88 ± 6.97	−0.563	0.575
Systolic BP in clinic	160.85 ± 9.76	157.80 ± 9.26	1.434	0.156
Diastolic BP in clinic	96.83 ± 12.43	95.18 ± 9.60	0.665	0.508
Preacupressure systolic BP	164.98 ± 16.72	154.80 ± 14.99	2.865	0.005
Preacupressure diastolic BP	96.33 ± 16.87	94.25 ± 12.42	0.627	0.533

*α* < 0.05. BP was measured in the right upper arm. BP, blood pressure; SD, standard deviation.

**Table 2:** Changes in blood pressure before and after acupressure in both groups.

Variables	Experimental group		Paired *t*	Control group		Paired *t*	
	(*n* = 40)			(*n* = 40)			
	Mean	SD		Mean	SD		
Systolic BP (mmHg)							
Before acupressure	165.0	16.72		154.8	14.99		
Immediately after acupressure	150.4	14.63	10.590^a*∗∗*^	153.7	15.55	1.732^a^	8.85^d*∗∗*^
15 min after acupressure	145.7	13.09	12.507^b*∗∗*^	154.8	15.75	0.000^b^	10.55^e*∗∗*^
30 min after acupressure	142.9	13.62	12.765^c*∗∗*^	155.7	16.21	−0.762^c^	10.98^f*∗∗*^
Diastolic BP (mmHg)							
Before acupressure	96.3	16.87		94.3	12.42		
Immediately after acupressure	92.7	16.16	4.216^a*∗∗*^	93.3	12.93	1.639^a^	2.61^d*∗∗*^
15 min after acupressure	90.8	15.37	6.824^b*∗∗*^	93.2	12.42	1.756^b^	4.40^e*∗∗*^
30 min after acupressure	88.6	15.09	6.799^c*∗∗*^	94.1	12.53	0.317^c^	5.79^f*∗∗*^

(1) a = (before acupressure)/(immediately after acupressure); b = (before acupressure/15 min after acupressure); c = (before acupressure/30 min after acupressure); d = experimental group/control group (immediately after acupressure − before acupressure); e = experimental group/control group (15 min after acupressure − before acupressure); f = experimental group/control group (30 min after acupressure − before acupressure). (2) *α* < 0.05; ^*∗*^ *p* < 0.05;  ^*∗∗*^ *p* < 0.01. BP, blood pressure.

**Table 3:** Linear mixed-effect model analysis of factors affecting systolic blood pressure change.

Variables	Estimation (*β*)	Standard error (SE)	*t*	*p* value	95% CI	
					Lower limit	Upper limit
*Y*-intercept	147.38	13.18	11.19	<0.001	121.14	173.63
Group						
Experimental/control group (control group = 0)	10.63	3.55	3.00	0.004	3.58	17.69
Gender						
Male/female (female = 0)	1.17	3.39	0.35	0.731	−5.58	7.92
Medication taken within last 8 h						
Yes/no (no = 0)	−3.27	3.56	−0.92	0.360	−10.36	3.81
Age	0.01	0.21	0.64	0.524	−0.28	0.55
30 min after *A*/before *A* (before *A* = 0)	0.90	1.44	0.62	0.533	−1.94	3.74
15 min after *A*/before *A* (before *A* = 0)	0.00	1.20	0.00	1.000	−2.36	2.36
Immediately after *A*/before *A* (before *A* = 0)	−1.13	0.86	−1.31	0.192	−2.82	0.57
Group *∗* time 3 (30 min after *A*/before *A*)	−23.03	2.04	−11.28	<0.001	−27.04	−19.01
Group *∗* time 2 (15 min after *A*/before *A*)	−19.25	1.69	−11.37	<0.001	−22.58	−15.92
Group *∗* time 1 (immediately after *A*/before *A*)	−13.50	1.22	−11.11	<0.001	−15.90	−11.11

Linear mixed-effect model for repeated measures; *α* < 0.05. time 1, time between preacupressure and immediately after acupressure; time 2, time between preacupressure and 15 min after acupressure; time 3, time between preacupressure and 30 min after acupressure. CI, confidence interval; *A*, acupressure.

**Table 4:** Linear mixed-effect model analysis of factors affecting diastolic blood pressure change.

Variables	Estimation (*β*)	SE	*t*	*p* value	95% CI	
					Lower limit	Upper limit
*Y*-intercept	124.43	10.60	11.74	<0.001	103.32	145.55
Group						
Experimental/control group (control group = 0)	0.27	2.83	0.10	0.923	−5.35	5.90
Gender						
Male/female (female = 0)	8.64	2.73	3.17	0.002	3.21	14.07
Medication taken within last 8 h						
Yes/no (no = 0)	−3.91	2.86	−1.36	0.177	−9.61	1.80
Age	−0.52	0.17	−3.14	0.002	−0.85	−0.19
30 min after *A*/before *A* (before *A* = 0)	−0.20	1.02	−0.20	0.845	−2.21	1.81
15 min after *A*/before *A* (before *A* = 0)	−1.08	0.84	−1.28	0.203	−2.73	0.58
Immediately after *A*/before *A* (before *A* = 0)	−0.93	0.60	−1.54	0.126	−2.11	0.26
Group *∗* time 3 (30 min after *A*/before *A*)	−7.53	1.44	−5.22	<0.001	−10.37	−4.69
Group *∗* time 2 (15 min after *A*/before *A*)	−4.48	1.19	−3.76	<0.001	−6.82	−2.13
Group *∗* time 1 (immediately after *A*/before *A*)	−2.68	0.85	−3.14	0.002	−4.35	−1.00

Linear mixed-effect model for repeated measures *α* < 0.05. Time 1, time between preacupressure and immediately after acupressure; time 2, time between preacupressure and 15 min after acupressure; time 3, time between preacupressure and 30 min after acupressure. *A*, acupressure; CI, confidence interval; SE, standard error.

## References

[1] World Health Organization (2016). *Global Health Observatory (GHO) Data: Raised Blood Pressure—Situation and Trends*.

[2] Gillespie C. D., Hurvitz K. A. (2013). Prevalence of hypertension and controlled hypertension—United States, 2007–2010. *MMWR Supplements*.

[3] Health Promotion Administration Ministry of Health and Welfare. 2007 survey on the prevalence of hypertension, hyperglycemia and hyperlipidemia in Taiwan. http://www.hpa.gov.tw/BHPNet/Web/HealthTopic/TopicArticle.aspx?id=201102140001&parentid=200712250011.

[4] Chobanian A. V., Bakris G. L., Black H. R. (2003). The seventh report of the joint national committee on prevention, detection, evaluation, and treatment of high blood pressure: the JNC 7 report. *Journal of the American Medical Informatics Association*.

[5] World Health Organization A global brief on hypertension. http://apps.who.int/iris/bitstream/10665/79059/1/WHO_DCO_WHD_2013.2_eng.pdf?ua=1.

[6] Huang Y. N., Wu T. Y., Kuo K. L. (2011). Prevalence, awareness, treatment and control of hypertension and diabetes mellitus among adults participating in health examinations in Taipei city. *Taiwan Journal of Family Medicine*.

[7] Taiwan Medical Association Hypertension treatment guidelines. http://mactin.com/zeno/web/zeno-med-file/HTN_guidelines.pdf.

[8] Shih S.-F., Lew-Ting C.-Y., Chang H.-Y., Kuo K. N. (2008). Insurance covered and non-covered complementary and alternative medicine utilisation among adults in Taiwan. *Social Science and Medicine*.

[9] Wang J., Xiong X. (2013). Evidence-based Chinese medicine for hypertension. *Evidence-Based Complementary and Alternative Medicine*.

[10] Chang L.-C., Huang N., Chou Y.-J., Lee C.-H., Kao F.-Y., Huang Y.-T. (2008). Utilization patterns of Chinese medicine and Western medicine under the National Health Insurance Program in Taiwan, a population-based study from 1997 to 2003. *BMC Health Services Research*.

[11] Chen F.-P., Chen T.-J., Kung Y.-Y. (2007). Use frequency of traditional Chinese medicine in Taiwan. *BMC Health Services Research*.

[12] Kaneko S., Watanabe M., Takayama S. (2013). Heart rate variability and hemodynamic change in the superior mesenteric artery by acupuncture stimulation of lower limb points: a randomized crossover trial. *Evidence-Based Complementary and Alternative Medicine*.

[13] Huang C. P., Lin J. C., Chen G. W., Li T. C., Chen M. F. (2004). Immediately decreased effect of blood pressure by acupuncture on essential hypertension. *Journal of Integrated Chinese and Western Medicine*.

[14] Wu H. L., Li X. Q., Wang X. (2008). The immediate effect on blood pressure of acupuncture at Taichong (LR 3) in 65 cases of hypertension patient with hyperactivity of liver-yang. *Journal of Traditional Chinese Medicine*.

[15] Hao P. Y., Wang X., Wen W. Q., Wu H. L. (2009). Clinical observation on 30 hypertension patients of liver yang hyperactivity treated by acupuncture at Taichong (LR 3). *Journal of Traditional Chinese Medicine*.

[16] Kim L.-W., Zhu J. (2010). Acupuncture for essential hypertension. *Alternative Therapies in Health and Medicine*.

[17] Yang D.-H. (2010). Effect of electroacupuncture on Quchi (LI 11) and Taichong (LR 3) on blood pressure variability in young patients with hypertension. *Zhongguo Zhen Jiu*.

[18] Chen Y. L. (2011). *Investigation the cardio-protective effects and molecular mechanisms of catgut embedding and electro acupuncture therapy in Taichong point of spontaneous hypertensive rats [Ph.D. dissertation]*.

[19] Wang J., Xiong X., Liu W. (2013). Acupuncture for essential hypertension. *International Journal of Cardiology*.

[20] Lai X. H., Wang J. Y., Nabar N. R. (2012). Proteomic response to acupuncture treatment in spontaneously hypertensive rats. *PLoS ONE*.

[21] Wang J.-Y., Tang C.-Z., He Z.-Q. (2011). Effect of moderate acupuncture-stimulation of ‘Taichong’ (LR 3) on blood pressure and plasma endothelin-1 levels in spontaneous hypertension rats. *Zhen Ci Yan Jiu*.

[22] Molassiotis A., Sylt P., Diggins H. (2007). The management of cancer-related fatigue after chemotherapy with acupuncture and acupressure: a randomised controlled trial. *Complementary Therapies in Medicine*.

[23] Xu C. H., Song G. M., Wang L. H., Chang M. C. (2007). The effect of acupoint massage on blood pressure in patients with hypertensive stroke. *Medical Journal of Qilu*.

[24] Kuritzky L. (2012). White coat hypertension: addressing the 10 most important questions. *Current Cardiology Reports*.

[25] Ma H.-W., Chang M.-L., Lin C.-J. (2007). A systematic review of acupressure for the application on nursing practice. *The Journal Nursing*.

[26] Faul F., Erdfelder E., Buchner A., Lang A.-G. (2009). Statistical power analyses using G^∗^Power 3.1: tests for correlation and regression analyses. *Behavior Research Methods*.

[27] Martins D., Nelson K., Pan D., Tareen N., Norris K. (2001). The effect of gender on age-related blood pressure changes and the prevalence of isolated systolic hypertension among older adults: data from NHANES III. *Journal of Gender-Specific Medicine*.

[28] Maranon R., Reckelhoff J. F. (2013). Sex and gender differences in control of blood pressure. *Clinical Science*.

[29] Kotsis V., Stabouli S., Pitiriga V., Toumanidis S., Papamichael C., Zakopoulos N. (2006). Ambulatory blood pressure monitoring and target organ damage: effects of age and sex. *Blood Pressure Monitoring*.

[30] Ji H., Zheng W., Wu X. (2010). Sex chromosome effects unmasked in angiotensin II-induced hypertension. *Hypertension*.

[31] Netea R. T., Thien T. (2004). Blood pressure measurement: we should all do it better!. *The Netherlands Journal of Medicine*.

[32] Bamia C., White I. R., Kenward M. G. (2013). Some consequences of assuming simple patterns for the treatment effect over time in a linear mixed model. *Statistics in Medicine*.

[33] Fontana A., Copetti M., Mazzoccoli G., Kypraios T., Pellegrini F. (2013). A linear mixed model approach to compare the evolution of multiple biological rhythms. *Statistics in Medicine*.

[34] Zhu G. Q., Su H. M., Ouyang L. X., Shu Z. H., Zhong X. Y. (2006). Research of needling Quchi and Taichong Points on treating hypertension patients with different syndrome. *Zhejiang Journal of Integrated Traditional Chinese and Western Medicine*.

[35] Wu Y.-H., Zhu G.-Q., Lin X.-Y., Oyang L. Y., Su H. M., Wu B. Q. (2004). Effect of needling quchi and taichong points on blood levels of endothelin and angiotension converting enzyme in patients with hypertension. *Zhongguo Zhong Xi Yi Jie He Za Zhi*.

[36] Lin J. G. Hypertention. *Zhong Xi Yi Bing Ming Dui Zhao da Ci Dian*.

[37] Wang B. Zhi-zhen-yao-lun; Su-wen. *Huangdi's Internal Classic*.

[38] Xiong X., Yang X., Liu W., Chu F., Wang P., Wang J. (2013). Trends in the treatment of hypertension from the perspective of traditional Chinese medicine. *Evidence-Based Complementary and Alternative Medicine*.

[39] Acupuncture.Com Liver 3. http://www.acupuncture.com/education/points/liver/liv3.htm.

